# Supplementary material for: The Effects of High CO2 and Strigolactones on Shoot Branching and Aphid–Plant Compatibility Control in Pea
Source: Int J Mol Sci. 2022 Oct 12;23(20):12160. doi: 10.3390/ijms232012160 (PMC9602761; doi:10.3390/ijms232012160)
Supplement: Supplementary file 1 [file ijms-23-12160-s001.zip › ijms-1903526-supplementary.pdf]

Supplementary Figures

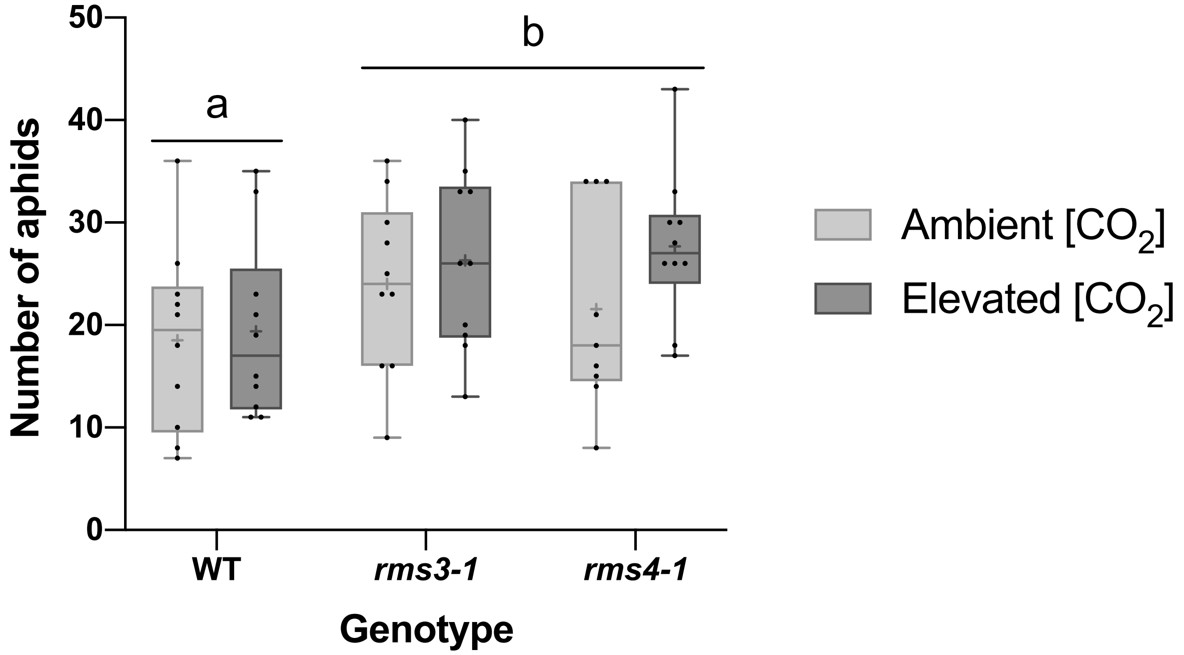

Figure S1: The effect of high CO_2 on aphid fecundity on wild type peas and mutants defective in strigolactone (SL) signalling (*rms3-1* and *rms4-1*). A single pea aphid nymph was placed on each 5-day old plant. Plants were then grown in air or under high [CO2]. Aphid numbers per plant were determined 15 days later. Ten replicates were performed for each SL mutant and wild type under each [CO_2]. Different letters indicate a statistically significant difference ($p \leq 0.05$) between wild type and strigolactone mutants (grouped).

Supplementary Tables

Table S1: Evaluation of normality and heteroscedasity using D'Agostino-Pearson omnibus K2 test and Spearman's test, respectively.

Dataset	D'Agostino-Pearson omnibus K2 test for normality of residuals		Spearman's test for heteroscedasticity		Subsequent statistical test(s)/ transformation performed
	**P-value**	**Passed (P > 0.05)?**	**P-value (one tailed)**	**Passed (P > 0.05)?**	
**Plant height**					
**Day 7**	0.3523	Yes	0.3844	Yes	Two-way ANOVA
**Day 14**	0.0741	Yes	0.1697	Yes	Two-way ANOVA
**Day 32**	0.7717	Yes	0.3627	Yes	Two-way ANOVA
**Total branching**					
**Day 7**	0.9927	Yes	0.0530	Yes	Two-way ANOVA
**Day 14**	0.0035	No	0.0029	No	Log transformation
**Day 14 log transformed**	0.2478	Yes	0.1549	Yes	Two-way ANOVA
**Day 32**	0.3399	Yes	<0.0001	No	Welch's t-test (CO_2); Welch's ANOVA (genotype)
**Biomass**					
**Day 28 dry weight**	0.0054	No	0.0004	No	Mann-Whitney (CO_2); Kruskal-Wallis (genotype)
**Day 28 fresh weight**	0.0023	No	0.0225	No	Mann-Whitney (CO_2); Kruskal-Wallis (genotype)
**Day 28 plant water content**	0.0995	Yes	0.3292	Yes	Two-way ANOVA
**Day 32 dry weight**	0.1875	Yes	0.0324	No	Welch's t-test (CO_2); Welch's ANOVA (genotype)
**Aphid fecundity**					
**Day 15; aCO_2 (Fig. 5)**	0.1956	Yes	0.3148	Yes	Unpaired t-test
**Day 15; aCO_2 & eCO_2 (Fig. S1)**	0.1290	Yes	0.1973	Yes	Unpaired t-test
**[Phytohormone]**					
**ACC**	0.3816	Yes	<0.0001	No	Welch's t-test (Aphid exposure); Welch's ANOVA (genotype)
**tZ**	0.0926	Yes	0.0001	No	Welch's t-test (Aphid exposure); Welch's ANOVA (genotype)
**iP**	0.2113	Yes	<0.0001	No	Welch's t-test (Aphid exposure); Welch's ANOVA (genotype)
**GA1**	0.1147	Yes	<0.0001	No	Welch's t-test (Aphid exposure); Welch's ANOVA (genotype)
**GA3**	<0.0001	No	0.0024	No	Mann-Whitney (Aphid exposure); Kruskal-Wallis (genotype)
**GA4**	0.9672	Yes	0.0613	Yes	Two-way ANOVA
**IAA**	0.4871	Yes	0.1459	Yes	Two-way ANOVA
**ABA**	0.1708	Yes	0.0100	No	Welch's t-test (Aphid exposure); Welch's ANOVA (genotype)
**JA**	0.0404	No	<0.0001	No	Log transformation
**JA log transformed**	0.8438	Yes	0.0482	No	Welch's t-test (Aphid exposure); Welch's ANOVA (genotype)
**SA**	0.3074	Yes	<0.0001	No	Welch's t-test (Aphid exposure); Welch's ANOVA (genotype)
**[Carbohydrate]**					
**Glucose**	0.9522	Yes	0.0683	Yes	Two-way ANOVA
**Fructose**	0.2311	Yes	0.0012	No	Welch's t-test (CO_2); Welch's ANOVA (genotype)
**Sucrose**	0.4158	Yes	0.1408	Yes	Two-way ANOVA
**Starch**	0.6199	Yes	0.0925	Yes	Two-way ANOVA

Table S2: The effect of the independent variables (genotype and [CO_2] or aphid exposure) on the response measured (dependent variable) were determined. *, < 0.05; **, < 0.01; ***, < 0.001; ****, < 0.0001.

Dependent variable	Test performed	Source of variation	% of total variation	P-value	P-value summary	Subsequent statistical test
**Plant height**						
**Day 7**						
	Two-way ANOVA	Interaction	38.99	<0.0001	****	
		Genotype	9.853	0.0606	ns	None
		[CO_2]	0.2606	0.6151	ns	None
**Day 14**						
	Two-way ANOVA	Interaction	3.573	0.5094	ns	
		Genotype	40.11	<0.0001	****	Tukey
		[CO_2]	2.833	0.1099	ns	None
**Day 32**						
	Two-way ANOVA	Interaction	0.06934	0.9372	ns	
		Genotype	92.09	<0.0001	****	Tukey
		[CO_2]	2.651	<0.0001	****	Sidak
**Total branching**						
**Day 7**						
	Two-way ANOVA	Interaction	3.175	0.4953	ns	
		Genotype	50.51	<0.0001	****	Tukey
		[CO_2]	0.08741	0.7598	ns	None
**Day 14**						
	Two-way ANOVA	Interaction	1.849	0.5503	ns	
		Genotype	67.41	<0.0001	****	Tukey
		[CO_2]	0.6997	0.2857	ns	None
**Day 32**						
	Welch's (one-way) ANOVA	Genotype	NA	<0.0001	****	Dunnett T3
	Welch's unpaired t-test	[CO_2]	NA	0.0044	**	Holm-Sidak
**Biomass**						
**Day 28 dry weight**						
	Kruskal-Wallis	Genotype	NA	0.3347	ns	None
	Mann-Whitney	[CO_2]	NA	0.0111	*	Holm-Sidak
**Day 28 fresh weight**						
	Kruskal-Wallis	Genotype	NA	0.3458	ns	None
	Mann-Whitney	[CO_2]	NA	0.0177	*	Holm-Sidak
**Day 28 plant water content**						
	Two-way ANOVA	Interaction	4.557	0.3332	ns	
		Genotype	51.40	<0.0001	****	Tukey
		[CO_2]	10.80	0.0019	**	Sidak
**Day 32 dry weight**						
	Welch's (one-way) ANOVA	Genotype	NA	0.0225	*	Dunnett T3
	Welch's unpaired t-test	[CO_2]	NA	0.0044	**	Holm-Sidak
**Aphid fecundity**						
**Day 15; aCO_2 (Fig. 5)**						
	Unpaired t-test	WT vs *rms* mutants (grouped)	NA	0.0077	**	Dunnett T3
**Day 15; aCO_2 & eCO_2 (Fig. S1)**						
	Unpaired t-test	WT vs *rms* mutants (grouped)	NA	0.0226	*	Dunnett T3
	Unpaired t-test	[CO_2]	NA	0.1873	ns	None
**[Phytohormone]**						
**ACC**						
	Welch's (one-way) ANOVA	Genotype	NA	0.8047	ns	None
	Welch's unpaired t-test	Aphid exposure	NA	<0.0001	****	Multiple unpaired t tests
**tZ**						

	Welch's (one-way) ANOVA	Genotype	NA	0.9809	ns	None
	Welch's unpaired t-test	Aphid exposure	NA	<0.0001	****	Holm-Sidak
**iP**						
	Welch's (one-way) ANOVA	Genotype	NA	0.8440	ns	None
	Welch's unpaired t-test	Aphid exposure	NA	<0.0001	****	Holm-Sidak
**GA1**						
	Welch's (one-way) ANOVA	Genotype	NA	0.7351	ns	None
	Welch's unpaired t-test	Aphid exposure	NA	<0.0001	****	Holm-Sidak
**GA3**						
	Kruskal-Wallis	Genotype	NA	0.0048	**	Dunn
	Mann-Whitney	Aphid exposure	NA	0.8153	ns	None
**GA4**						
	Two-way ANOVA	Interaction	4.079	0.0010	**	
		Genotype	3.369	0.0024	**	Sidak
		Aphid exposure	89.86	<0.0001	****	Sidak
**IAA**						
	Two-way ANOVA	Interaction	15.38	0.2105	ns	
		Genotype	4.425	0.6208	ns	None
		Aphid exposure	0.1441	0.8603	ns	None
**ABA**						
	Welch's (one-way) ANOVA	Genotype	NA	0.5007	ns	None
	Welch's unpaired t-test	Aphid exposure	NA	0.9585	ns	None
**JA**						
	Welch's (one-way) ANOVA	Genotype	NA	0.5554	ns	None
	Welch's unpaired t-test	Aphid exposure	NA	<0.0001	****	Holm-Sidak
**SA**						
	Welch's (one-way) ANOVA	Genotype	NA	0.0324	ns	Dunnett's T3 test
	Welch's unpaired t-test	Aphid exposure	NA	0.2973	ns	None
**[Carbohydrate]**						
**Glucose**						
	Two-way ANOVA	Interaction	1.923	0.9090	ns	
		Genotype	2.146	0.8913	ns	None
		[CO_2]	56.78	<0.0001	****	Sidak
**Fructose**						
	Welch's (one-way) ANOVA	Genotype	NA	0.8688	ns	None
	Welch's unpaired t-test	[CO_2]	NA	0.0009	***	Holm-Sidak
**Sucrose**						
	Two-way ANOVA	Interaction	1.462	0.7397	ns	
		Genotype	4.355	0.2473	ns	Tukey
		[CO_2]	79.41	<0.0001	****	Sidak
**Starch**						
	Two-way ANOVA	Interaction	0.7123	0.5559	ns	
		Genotype	0.8778	0.4551	ns	None
		[CO_2]	93.80	<0.0001	****	Sidak

Table S3: Multiple comparison tests to compare **genotype** means. *, < 0.05; **, < 0.01; ***, < 0.001; ****, < 0.0001.

Dependent variable	Multiple comparison test	Comparison	Adjusted P-value	Summary
**Plant height**				
**Day 14**	Tukey			
		WT vs. *rms3-1*	0.0018	**
		WT vs. *rms4-1*	<0.0001	****
		WT vs. *rms1-2*	<0.0001	****
		WT vs. *rms5-3*	0.0011	**
		rms3-1 vs. *rms4-1*	0.6345	ns
		rms3-1 vs. *rms1-2*	0.8457	ns
		rms3-1 vs. *rms5-3*	0.9998	ns
		rms4-1 vs. *rms1-2*	0.9956	ns
		rms4-1 vs. *rms5-3*	0.7324	ns
		rms1-2 vs. *rms5-3*	0.9108	ns
**Day 32**	Tukey			
		WT vs. *rms3-1*	<0.0001	****
		WT vs. *rms4-1*	<0.0001	****
		WT vs. *rms1-2*	<0.0001	****
		WT vs. *rms5-3*	<0.0001	****
		rms3-1 vs. *rms4-1*	0.9981	ns
		rms3-1 vs. *rms1-2*	0.9500	ns
		rms3-1 vs. *rms5-3*	0.9932	ns
		rms4-1 vs. *rms1-2*	0.8422	ns
		rms4-1 vs. *rms5-3*	>0.9999	ns
		rms1-2 vs. *rms5-3*	0.7809	ns
**Total branching**				
**Day 7**	Tukey			
		WT vs. *rms3-1*	0.1843	ns
		WT vs. *rms4-1*	0.0002	***
		WT vs. *rms1-2*	0.1752	ns
		WT vs. *rms5-3*	0.3999	ns
		rms3-1 vs. *rms4-1*	0.1047	ns
		rms3-1 vs. *rms1-2*	0.0004	***
		rms3-1 vs. *rms5-3*	0.9905	ns
		rms4-1 vs. *rms1-2*	<0.0001	****
		rms4-1 vs. *rms5-3*	0.0353	*
		rms1-2 vs. *rms5-3*	0.0017	**
**Day 14**	Tukey			
		WT vs. *rms3-1*	<0.0001	****
		WT vs. *rms4-1*	<0.0001	****
		WT vs. *rms1-2*	<0.0001	****
		WT vs. *rms5-3*	<0.0001	****
		rms3-1 vs. *rms4-1*	0.0963	ns
		rms3-1 vs. *rms1-2*	0.9061	ns
		rms3-1 vs. *rms5-3*	0.0032	**
		rms4-1 vs. *rms1-2*	0.4573	ns
		rms4-1 vs. *rms5-3*	0.7064	ns
		rms1-2 vs. *rms5-3*	0.0368	*
**Day 32**	Dunnett T3			
		WT vs. *rms3-1*	<0.0001	****
		WT vs. *rms4-1*	<0.0001	****
		WT vs. *rms1-2*	<0.0001	****
		WT vs. *rms5-3*	<0.0001	****
		rms3-1 vs. *rms4-1*	0.9822	ns
		rms3-1 vs. *rms1-2*	>0.9999	ns
		rms3-1 vs. *rms5-3*	0.9194	ns

		rms4-1 vs. *rms1-2*	0.9582	ns
		rms4-1 vs. *rms5-3*	>0.9999	ns
		rms1-2 vs. *rms5-3*	0.8856	ns
**Biomass**				
**Day 28 plant water content**	Tukey			
		WT vs. *rms3-1*	<0.0001	****
		WT vs. *rms4-1*	<0.0001	****
		WT vs. *rms1-2*	0.0003	***
		WT vs. *rms5-3*	0.0014	**
		rms3-1 vs. *rms4-1*	0.9025	ns
		rms3-1 vs. *rms1-2*	0.4055	ns
		rms3-1 vs. *rms5-3*	0.1678	ns
		rms4-1 vs. *rms1-2*	0.8631	ns
		rms4-1 vs. *rms5-3*	0.5566	ns
		rms1-2 vs. *rms5-3*	0.9864	ns
**Day 32 dry weight**	Dunnett T3			
		WT vs. *rms3-1*	>0.9999	ns
		WT vs. *rms4-1*	0.3580	ns
		WT vs. *rms1-2*	0.6315	ns
		WT vs. *rms5-3*	>0.9999	ns
		rms3-1 vs. *rms4-1*	0.0762	ns
		rms3-1 vs. *rms1-2*	0.3352	ns
		rms3-1 vs. *rms5-3*	>0.9999	ns
		rms4-1 vs. *rms1-2*	>0.9999	ns
		rms4-1 vs. *rms5-3*	0.0681	ns
		rms1-2 vs. *rms5-3*	0.3324	ns
**Aphid fecundity**				
**Day 15; aCO_2 (Fig. 5)**	Dunnett T3			
		WT vs. *rms3-1*	0.0228	*
		WT vs. *rms4-1*	0.1173	ns
		WT vs. *rms2-1*	0.6800	ns
		WT vs. *rms5-3*	0.3022	ns
**Day 15; aCO_2 & eCO_2 (Fig. S1)**	Dunnett T3			
		WT vs. *rms3-1*	0.0537	ns
		WT vs. *rms4-1*	0.0867	ns
**Phytohormone [ ]**				
**GA3**	Dunn			
		WT vs. *rms3-1*	0.0778	ns
		WT vs. *rms4-1*	0.0044	**
		rms3-1 vs. *rms4-1*	>0.9999	ns
**GA4**	Sidak			
		WT vs. *rms3-1*	0.0088	**
		WT vs. *rms4-1*	0.0050	**
		rms3-1 vs. *rms4-1*	0.9564	ns
**SA**	Dunnett's T3 test			
		WT vs. *rms3-1*	0.4020	ns
		WT vs. *rms4-1*	0.0479	*
		rms3-1 vs. *rms4-1*	0.3310	ns

Table S4: Multiple comparison tests to compare means between ambient and elevated **[CO_2] within genotype**. *, < 0.05; **, < 0.01; ***, < 0.001; ****, < 0.0001.

Dependent variable	Multiple comparison test	Comparison	Adjusted P-value	Summary
**Plant height**				
**Day 32**	Sidak	Ambient [CO_2] - Elevated [CO_2]		
		WT	0.0202	*
		rms3-1	0.0406	*
		rms4-1	0.3339	ns
		rms1-2	0.0960	ns
		rms5-3	0.0781	ns
**Total branching**				
**Day 32**	Holm-Sidak	Ambient [CO_2] - Elevated [CO_2]		
		WT	0.0004	***
		rms3-1	0.0609	ns
		rms4-1	0.0601	ns
		rms1-2	0.0302	*
		rms5-3	0.0779	ns
**Biomass**				
**Day 28 dry weight**	Holm-Sidak	Ambient [CO_2] - Elevated [CO_2]		
		WT	0.4785	ns
		rms3-1	0.2069	ns
		rms4-1	0.2951	ns
		rms1-2	0.6507	ns
		rms5-3	0.7369	ns
**Day 28 fresh weight**	Holm-Sidak	Ambient [CO_2] - Elevated [CO_2]		
		WT	0.7162	ns
		rms3-1	0.4887	ns
		rms4-1	0.4887	ns
		rms1-2	0.7355	ns
		rms5-3	0.7355	ns
**Day 28 plant water content**	Sidak	Ambient [CO_2] - Elevated [CO_2]		
		WT	0.0212	*
		rms3-1	0.2230	ns
		rms4-1	0.9933	ns
		rms1-2	0.9234	ns
		rms5-3	0.8572	ns
**Day 32 dry weight**	Holm-Sidak	Ambient [CO_2] - Elevated [CO_2]		
		WT	0.0031	**
		rms3-1	0.0011	**
		rms4-1	0.0099	**
		rms1-2	0.0098	**
		rms5-3	0.0015	**
**Carbohydrate [ ]**				
**Glucose**	Sidak	Ambient [CO_2] - Elevated [CO_2]		
		WT	0.0804	ns
		rms3-1	0.0918	ns
		rms4-1	0.0457	*
		rms1-2	0.5064	ns
		rms5-3	0.1174	ns
**Fructose**	Holm-Sidak	Ambient [CO_2] - Elevated [CO_2]		
		WT	0.0376	*
		rms3-1	0.0530	ns
		rms4-1	0.0026	**
		rms1-2	0.0530	ns
		rms5-3	0.0075	**
**Sucrose**	Sidak	Ambient [CO_2] - Elevated [CO_2]		

		WT	0.0004	***
		rms3-1	0.0003	***
		rms4-1	0.0002	***
		rms1-2	0.0016	**
		rms5-3	0.0102	*
**Starch**	Sidak	Ambient [CO_2] - Elevated [CO_2]		
		WT	<0.0001	****
		rms3-1	<0.0001	****
		rms4-1	<0.0001	****
		rms1-2	<0.0001	****
		rms5-3	<0.0001	****

Table S5: Multiple comparison tests to compare means between aphid vs no **aphid exposure within genotype**. *, < 0.05; **, < 0.01; ***, < 0.001; ****, < 0.0001.

**Dependent variable**	**Multiple comparison test**	**Comparison**	**Adjusted P-value**	**Summary**
**Phytohormone [ ]**				
**ACC**	Holm-Sidak	Control - Aphid exposed		
		WT	0.0154	*
		rms3-1	0.0082	**
		rms4-1	0.0154	*
**tZ**	Holm-Sidak	Control - Aphid exposed		
		WT	0.0028	**
		rms3-1	0.0016	**
		rms4-1	0.0028	**
**iP**	Holm-Sidak	Control - Aphid exposed		
		WT	0.0779	ns
		rms3-1	0.0452	*
		rms4-1	0.0779	ns
**GA1**	Holm-Sidak	Control - Aphid exposed		
		WT	0.0191	*
		rms3-1	0.0203	*
		rms4-1	0.0332	*
**GA4**	Sidak	Control - Aphid exposed		
		WT	<0.0001	****
		rms3-1	<0.0001	****
		rms4-1	<0.0001	****
**JA**	Holm-Sidak	Control - Aphid exposed		
		WT	<0.0001	****
		rms3-1	0.0001	***
		rms4-1	0.0006	***

Table S6: The correlation of aphid numbers to phytohormones in pea on which a single *Acyrthosiphon pisum* nymph was allowed to reproduce for a period of 15 days.

**Hormone**	**Pearson correlation coefficient (*r*)**	**p-value (Pearson)**	**Coefficient of determination (R^2)**
**ACC**	-0.161	0.395	0.026
**tZ**	-0.323	0.081	0.105
**iP**	0.103	0.588	0.011
**GA1**	0.014	0.942	0.000
**GA3**	**-0.429**	**0.018**	**0.184**
**IAA**	-0.186	0.326	0.034
**ABA**	0.024	0.901	0.001
**JA**	-0.328	0.077	0.107
**SA**	0.061	0.748	0.004
**GA4**	0.245	0.192	0.060
